# High dose vitamin D supplementation does not affect biochemical bone markers in multiple sclerosis – a randomized controlled trial

**DOI:** 10.1186/s12883-017-0851-0

**Published:** 2017-04-04

**Authors:** Trygve Holmøy, Jonas Christoffer Lindstrøm, Erik Fink Eriksen, Linn Hofsøy Steffensen, Margitta T. Kampman

**Affiliations:** 1 Department of Neurology, Akershus University Hospital, Lørenskog, Norway; 2 Institute of Clinical Medicine, University of Oslo, Oslo, Norway; 3 Helse Øst Health Services and Research Centre, Akershus University Hospital, Lørenskog, Norway; 4 Department of Endocrinology, Oslo University Hospital, Oslo, Norway; 5 Department of Neurology, University Hospital of North Norway, Tromsø, Norway; 6 Department of Clinical Medicine, University of Tromsø, Tromsø, Norway

**Keywords:** Multiple sclerosis, vitamin D, osteoporosis, randomized controlled trial

## Abstract

**Background:**

People with multiple sclerosis have high risk of osteoporosis and fractures. A poor vitamin D status is a risk factor for MS, and vitamin D supplementation has been recommended both to prevent MS progression and to maintain bone health.

**Methods:**

We assessed the effect of 20,000 IU vitamin D_3_ weekly compared to placebo on biochemical markers of bone metabolism in 68 persons with relapsing remitting multiple sclerosis.

**Results:**

Serum levels of 25-hydroxyvitamin D more than doubled in the vitamin D group, and parathyroid hormone decreased in the vitamin D group compared to the placebo group at week 48 and week 96. There was however no effect on bone formation as measured by procollagen type I N propeptide (PINP), or on bone resorption as measured by C-terminal cross-linking telopeptide of type I collagen (CTX1). Neither PINP nor CTX1 predicted bone loss from baseline to week 96.

**Conclusions:**

These findings corroborate the previously reported lack of effect of weekly high dose vitamin D supplementation on bone mass density in the same patients, and suggest that such vitamin D supplementation does not prevent bone loss in persons with MS who are not vitamin D deficient.

**Trial registration:**

The trial was registered at ClinicalTrials.gov on April 4 2008, registration number NCT00785473.

## Background

Low levels of vitamin D are associated with increased future risk of developing multiple sclerosis and with increased disease activity [1–3]. Vitamin D is also essential for bone health. Several studies have shown that people with multiple sclerosis (MS) are at increased risk of developing osteoporosis [4, 5]. Physical disability is likely the main driver of accelerated bone loss in MS, but also disease duration and lifetime steroid dose are associated with low bone mineral density (BMD) [4, 6]. Low BMD is however prevalent also in ambulatory persons with MS even shortly after clinical onset [6, 7], suggesting that shared etiological factors such as low vitamin D may operate in both MS and osteoporosis.

The combination of osteoporosis and high risk of falling may add to the burden of disease through increased risk of fractures. In line with this, large population based studies have shown that persons with MS have a marked increase of fractures compared to the general population [8–12]. Data from the Danish MS Registry and The National Hospital Discharge Registry showed that the risk of fractures of tibia, hip and femur in persons with MS was three to six times higher than in the general population [10].

Although the role of vitamin D supplementation on disease activity in MS is unclear [13], several authors have suggested that vitamin D should be monitored to prevent osteoporosis and fractures [5, 14–16]. The optimal intake of vitamin D and serum level of 25-hydroxyvitamin D is however controversial. Whereas the National Institute of Medicine considers a serum level of 25-hydroxyvitamin (25(OH)D) at 50 nmol/L and a daily intake of 600 IU vitamin D adequate for the general population [17], others argue that the serum level needed for both optimal bone health and for the potentially beneficial non-calcemic effects is at least 75 nmol/L [18–20]. There is, however limited evidence on the effect of vitamin D supplementation on bone heath in MS.

We have previously reported a randomized controlled trial (RCT) of weekly supplementation with 20,000 IU vitamin D_3_ compared to placebo in fully ambulatory (expanded disability status scale ≤4.5) persons with relapsing remitting MS living above the Arctic Circle [21]. This dose has proven safe in several RTCs in the same area [22]. Even though people with MS may need more vitamin D than others to reach the same 25-hydroxyvitamin D (25(OH)D) serum concentration, [23], we expected that this dose would bring the vast majority of patients to 25(OH)D levels considered optimal for bone health and also within the range associated with decreased disease activity. Although bone mineral density (BMD) decreased significantly in the placebo group and not in the vitamin D treated group, the primary outcome (difference in percentage change in BMD between groups) was negative [21].

The markers of bone formation procollagen type I N propeptide (PINP) and bone resorption C-terminal cross-linking telopeptide of type I collagen (CTX1) have been shown to predict fracture risk and to reflect the response to osteoporosis treatment [24]. These markers are recommended as reference markers in observational and treatment studies in osteoporosis by the International Osteoporosis Foundation and the International Federation of Clinical Chemistry and Laboratory Medicine [25], and could be more sensitive for treatment effects than BMD. The aim of the current study was to examine if CTX and PINP predict bone loss, and if vitamin D_3_ supplementation affect these markers of bone formation and turnover in persons with MS.

## Methods

The design of the RCT have been reported previously [21, 26]. Briefly, 71 RRMS patients from Troms and Finnmark (the northernmost counties in Norway), aged 18–50 years and with Kurtzke’s Expanded Disability Status Scale (EDSS) score ⩽4.5 were included in the original study [21]. The exclusion criteria comprised a history of conditions or diseases affecting bone, pregnancy or lactation the past 6 months, use of bone-active medications other than intravenous methylprednisolone for treatment of MS relapses, a history of nephrolithiasis during the previous 5 years, or menopause.

The participants were randomized to receive either once-weekly oral 20,000 IU vitamin D_3_ (Dekristol™; SWISS CAPS AG, Kirchberg, Switzerland) or placebo. All participants also received 500 mg calcium daily (calcium carbonate, Weifa AS, Oslo, Norway). Participants who had gastrointestinal side effects attributed to Weifa calcium switched to Calcium Sandoz™ effervescent tablets (calcium lactate-gluconate and calcium carbonate, Sandoz A/S, Odense, Denmark), or discontinued the calcium supplement if their estimated dietary calcium intake as measured by a validated food frequency questionnaire exceeded 800 mg/day [27]. By capsule count, all subjects were ≥80% (mean 98%, range 80–100%) adherent to the study medication [21].

Measurement of BMD at the hip (mean of left and right), the spine (anterior–posterior spine L1–L4), and the non-dominant ultra-distal radius by DXA (dual X-ray absorptiometry) was performed by trained technicians at the University Hospital of North-Norway, using a Lunar Prodigy advanced densitometer (Lunar Radiation Corp., Madison, WI, USA.). The long-term precision was 0.26–0.28%, obtained by daily calibration of the densitometer. One fourth of the patients had low BMD (z-scores blow −2) at baseline [6].

Serum samples were collected at baseline (January or February for all participants) and at week 48 and 96 (randomly to intake of vitamin D supplementation), and frozen at −70 °C until batch analyses. 25(OH)D was measured by spectroscopy detecting total concentrations of both 25(OH)D_2_ and 25(OH)D_3_ at the Hormone laboratory at Haukeland University Hospital. The coefficient of variat4ion (CV) was 5.3% at 20 nmol/L and 4.0% at 239 nmol/L 25(OH)D. PINP and CTX1 were measured by electrochemiluminiscence at the Hormone laboratory at Oslo University Hospital. The CVs were 11% for PINP and 12% for CTX1, and the detection limits were 5–1200 μg/l and 0,07–6,00 μg/l respectively.

The associations between bone markers and BMD at baseline, and whether the concentrations of PINP and CTX1 at baseline predicted BMD change from baseline to week 96, were analyzed with linear regression. The longitudinal changes in CTX1, PINP and PTH were modelled with two separate linear mixed models. The models included time of measurement, treatment arm, time-treatment interaction, and a random intercept for each participant. The markers were log-transformed, making the estimated differences interpretable as percentages. The models formed the basis for all inferences on the relationship between vitamin D and the markers. To investigate whether disease modifying drugs could influence the results, we also used a model which included the drug treatment status at each sample time.

## Results

Serum samples for measurement of bone markers were available from 68 participants who completed the study. Baseline characteristics of the study population are shown in Table 1. As reported previously [28] there were no group differences in age, body mass index (BMI), smoking, use of disease modifying drugs, disability as measured by EDSS, calcium intake or relapse rate the previous year. The serum concentration of 25(OH)D increased to 123.2 ± 34.2 nmol/L at week 96 in the vitamin D group, and to 61.8 ± 25.2 nmol/L in the placebo group. Ionized calcium was similar and unchanged at baseline and week 96 in both groups (1.2 ± 0.0 nmol/L).

**Table 1:** Baseline characteristics

		Vitamin D group (*N* = 35)	Placebo group (*N* = 33)
Females	N (%)	24 (69)	24 (73)
Age (years)	Mean (range)	40 (21–50)	41 (26–50)
Body mass index	Mean (range)	25.9 (21.0–40.7)	26.4 (18.4–39.9)
Ongoing smoking	N (%)	15 (43)	14 (42)
EDSS	Median (range)	2.5 (0–4.5)	2.0 (0–4.5)
Annualised relapse rate	Mean (range)	0.11 (0–0.54)	0.15 (0–1.10)
Immunomodulatory treatment	N (%)	17 (49)^a^	17 (52)^b^
Serum 25(OH)D (nmol/L)	Mean (SD)	55.6 (29.0)	57.3 (21.8)
Hip BMD (mg/cm^2^)	Mean (SD)	1018.8 (98.8)	968.9 (119.9)
Spine BMD (mg/cm^2^)	Mean (SD)	1205.2 (117.7)	1165.7 (135.6)
Distal radius BMD (mg/cm^2^)	Mean (SD)	484.8 (67.1)	472.8 (80.6)

^a^16 patients on IFN-β and one on glatiramer acetate

^b^15 patients on IFN-β, one on glatiramer acetate and one on natalizumab

The concentrations of CTX1, PINP and PTH did not differ significantly between the groups at baseline (Table 2). The concentrations of CTX1 and PINP remained similar between treatment groups throughout the study group, whereas PTH was lower in the vitamin D group at both week 48 and at week 96.

**Table 2:** Bone markers and PTH throughot the study period

	CTX1 (μg/l)			PINP (μg/l)			PTH pmol/L		
	Placebo	Vitamin D	*p*-value*	Placebo	Vitamin D	*p*-value*	Placebo	Vitamin D	*p*-value*
Baseline. mean (SD)	0.20 (0.10)	0.22 (0.11)	0.59	43.10 (15.1)	40.32 (10.0)	0.57	4.75 (1.08)	4.68 (1.29)	0.66
Week 48. mean (SD)	0.22 (0.16)	0.21 (0.11)	0.79	43.36 (17.2)	38.56 (10.6)	0.43	3.68 (1.04)	3.13 (0.96)	0.017
Week 96. mean (SD)	0.23 (0.17)	0.23 (0.12)	0.98	42.54 (15.0)	43.52 (10.6)	0.22	3.96 (1.27)	3.39 (1.00)	0.046

*Obtained from linear mixed model

The associations between the bone markers and BMD are presented in Table 3. There was a weak negative association between CTX1 and PINP and hip BMD at baseline, and between CTX1 and spine BMD at baseline (*p* < 0.05). The baseline marker concentrations did not predict change in BMD from baseline to week 48 or week 96.

**Table 3:** Association between bone markers at baseline and BMD (regression coefficients)

BMD	CTX1	PINP
Baseline hip	−279.4*	−2.42*
Baseline spine	−385.0*	−2.12
Baseline distal radius	91.3	1.05
DELTA hip	−7.6	0.10
DELTA spine	61.5	0.40
DELTA distal radius	−30.9	0.30

*p < 0.05; DELTA indicated the difference in BMD from baseline to week 96

The effect of vitamin D supplementation was finally analyzed using a linear mixed model for each bone marker with random patient-wise intercepts. There was no significant difference between treatment groups in the change of CTX1, PINP or PTH from baseline to week 48 or from baseline to week 96 (Table 4). These results did not change substantially when immune modulatory treatment was included in the model (results not shown). In total 14 patients received methylprednisolone for MS attack. Of these two in the placebo and two in the vitamin D groups were treated during the last 6 months prior to the first blood sampling, and three in the vitamin D group and one in the placebo group during the last 6 months prior to the last blood sampling. Excluding the 14 patients treated with methylprednisolone did not alter the results (data nor shown).

**Table 4:** Effect of high dose vitamin D supplementation compared to placebo on bone markers

	Week 48		Week 96	
	Change from baseline, percent difference (95% CI)	*p*-value*	Change from baseline, percent difference (95%CI)	*p*-value*
PINP	-5.10% (−17.77, 7.56)	0.43	10.26% (−2.48 22.99)	0.12
CTX1	-6.68% (−33.62, 20.25)	0.63	−3.60% (−30.67, 23.48)	0.80
PTH	-13.69% (−29.55, 2.12)	0.09	−10.9% (−26.81, 5.03)	0.17

*Obtained from linear mixed model

## Discussion

To our knowledge the effect on vitamin D supplementation on markers of bone formation and resorption in persons with MS has not been reported previously. We found that increasing mean 25(OH)D levels from 56 to 123 nmol/L with weekly high dose vitamin D supplementation did not influence biochemical markers of bone formation or turnover in persons with MS receiving calcium supplementation. This concurs with the previously negative results on BMD in the same cohort [21], and also with data obtained in healthy persons [29]. Moreover, we here showed that neither CTX1 nor PINP at baseline predicted BMD loss the subsequent 96 weeks. This is in contrast with a previous study comprising 29 MS patients followed for 3.1 ± 1.9 years, reporting a decline in BMD in the hip but not in the lumbal spine correlated inversely with bone turnover markers [30]. Whereas the patients included in these studies were fairly comparable regarding disease duration, BMI and disability levels, all known to be important for bone health in MS, only 50% of our patients received immunomodulatory treatment compared to 100% in the previous study [30]. Immunomodulatory drugs, including interferon beta which was most commonly used by our patients, could affect bone loss [31]. The use of immunomodulatory treatment did however not influence the effect of vitamin D on bone markers, and was not associated with BMD at baseline in our patients [32]. Other possible explanations for this discrepancy include differences in sample sizes and duration of follow up.

The vitamin D measurements in this study were performed in January and February and should therefore represent the seasonal nadir fairly well [33]. At this time point 18 of 35 patients in the treatment group had 25(OH)D levels above 50 nmol/L, which are considered adequate for maintenance of good bone health by the Institute of Medicine. Clear evidence of vitamin D deficiency (25(OH)D below 25 nmol) were only recorded in nine patients in each treatment group. It is conceivable that people with vitamin deficiency have a better effect of vitamin D supplementation on bone health than people with adequate vitamin D status, and that the low proportion of patients with vitamin D deficiency contributed to the negative results.

RCTs of vitamin D supplementation have not shown a consistent effect on BMD or fracture risk in the general population [17]. This does not exclude that particular subgroups with increased risk of osteoporosis due to immobilization, inadequate nutrition, medication or disease may need vitamin D supplementation to maintain bone health [8]. Our study population had rather low disease activity and their ambulation was only moderately impaired. MS patients with more advanced disability are more prone to both accelerated bone loss and vitamin D deficiency D [34], and could benefit more from vitamin D supplementation than those included in this study.

There are several strengths and limitations of this study. The randomized design minimized the risk of selection bias, and rigorous follow-up throughout the study period ensured adherence to the study medication. The optimal 25(OH)D level for bone health is not known, but the dose used in this trial was well above 800 IU per day which has been suggested to prevent fracture in meta-analysis [35], and brought 25(OH)D in most patients above 75–100 nmol/L which has been suggested by several experts to be adequate [19, 36]. The main weakness of the study is the limited size, which was not sufficient to perform subgroup analyses or to detect minor yet relevant effects of vitamin D supplementation. Moreover, patients were allowed to continue use of vitamin D supplements, and more than 50% of the patients in the placebo group reported a vitamin D intake exceeding 7.5 μg/day. This concurs with the generally favorable vitamin D status of our patients. It is conceivable that depriving the patients from their vitamin D supplements could have increased the chance for a positive result, but it would expose patients in the placebo group to the risks of vitamin D deficiency, and would be particularly problematic in a population living north of the Arctic Circle. Another potential weakness is the use of weekly dosing of vitamin D3. Although weekly dosing leads to a stable serum concentration of 25(OH)D, which has a long half- life, the effect on other vitamin D metabolites is different. Notably, the serum concentration of native vitamin D, which likely plays an important role as substrate for synthesis of active 1,25-dihydroxyvitamin D in several tissues, peaks after six to 8 h and thereafter falls rapidly [37]. It is therefore possible that daily supplementation is better than weekly supplementation of vitamin D.

## Conclusions

Our results do not support that high dose weekly vitamin D supplementation is beneficial for bone health in ambulatory persons with MS, and suggest that weekly vitamin D supplementation alone is not sufficient to prevent bone loss in persons with MS who are not vitamin D deficient.

## Abbreviations

25(OH)D: 25-hydroxyvitamion D
BMD: Bone mineral density
CTX1: C-terminal cross-linking telopeptide of type I collagen
CV: Coefficient of variation
MS: Multiple sclerosis
PINP: Procollagen type 1 N-terminal propeptide
PTH: Parathyroid hormone
